# Immunomodulatory Effects of the *Agaricus blazei* Murrill-Based Mushroom Extract AndoSan in Patients with Multiple Myeloma Undergoing High Dose Chemotherapy and Autologous Stem Cell Transplantation: A Randomized, Double Blinded Clinical Study

**DOI:** 10.1155/2015/718539

**Published:** 2015-01-18

**Authors:** Jon-Magnus Tangen, Anne Tierens, Jo Caers, Marilene Binsfeld, Ole Kristoffer Olstad, Anne-Marie Siebke Trøseid, Junbai Wang, Geir Erland Tjønnfjord, Geir Hetland

**Affiliations:** ^1^Deparment of Haematology, Oslo University Hospital, 0424 Oslo, Norway; ^2^Institute of Clinical Medicine, University of Oslo, 0372 Oslo, Norway; ^3^Department of Pathology, Oslo University Hospital, 0424 Oslo, Norway; ^4^Laboratory Medicine Program, Toronto General Hospital, University Health Network, Toronto, ON, Canada M5G 2C4; ^5^Laboratory of Hematology, GIGA-Research, University of Liege, 4000 Sart Tilman, Belgium; ^6^Department Medical Biochemistry, Oslo University Hospital, 0424 Oslo, Norway; ^7^Department of Immunology and Transfusion Medicine, Oslo University Hospital, 0424 Oslo, Norway

## Abstract

Forty patients with multiple myeloma scheduled to undergo high dose chemotherapy with autologous stem cell support were randomized in a double blinded fashion to receive adjuvant treatment with the mushroom extract AndoSan, containing 82% of *Agaricus blazei* Murrill (19 patients) or placebo (21 patients). Intake of the study product started on the day of stem cell mobilizing chemotherapy and continued until the end of aplasia after high dose chemotherapy, a period of about seven weeks. Thirty-three patients were evaluable for all study endpoints, while all 40 included patients were evaluable for survival endpoints. In the leukapheresis product harvested after stem cell mobilisation, increased percentages of Treg cells and plasmacytoid dendritic cells were found in patients receiving AndoSan. Also, in this group, a significant increase of serum levels of IL-1ra, IL-5, and IL-7 at the end of treatment was found. Whole genome microarray showed increased expression of immunoglobulin genes, Killer Immunoglobulin Receptor (KIR) genes, and HLA genes in the *Agaricus* group. Furthermore, AndoSan displayed a concentration dependent antiproliferative effect on mouse myeloma cells *in vitro*. There were no statistically significant differences in treatment response, overall survival, and time to new treatment. The study was registered with Clinicaltrials.gov NCT00970021.

## 1. Introduction

Multiple myeloma is a neoplastic disorder caused by malignant transformation of plasma cells. The main clinical features are bone marrow failure, lytic bone lesions, and renal insufficiency. The age adjusted incidence of multiple myeloma is 4/100 000 and median age at diagnosis is 70 years [1]. High dose chemotherapy with autologous stem cell support is standard first line treatment for patients below the age of 65–70 years, taking into consideration patients' general condition and comorbidity [2]. The majority responds well to the initial treatment, but all patients eventually relapse. In recent years, the introduction of three new drugs, thalidomide, bortezomib, and lenalidomide, has significantly improved the treatment results, but multiple myeloma remains an incurable disease. In patients below 65 years of age, 10 years survival is currently estimated to about 30% [3].

*Agaricus blazeii *Murrill (AbM) is an edible Basidiomycetes mushroom, which grows naturally in Piedade outside Sao Paulo in Brazil. It is widely used locally as a food supplement and as treatment for various medical conditions [4]. In the 1960s, AbM was brought to Japan for industrial cultivation and this became the starting point for scientific research. The fruiting body of AbM is rich in *β*-glucans, which is a potent stimulant of the innate immune system. *β*-Glucan from AbM has been shown to have an antitumor effect both* in vitro* (fibrosarcoma [5], ovarian cancer [6], hepatocarcinoma [7], and leukemia cells [8]) and in animal models (fibrosarcoma [9], multiple myeloma [10], and lung cancer [11]). It has therefore been assumed that the medicinal effect of AbM is mainly due to the immunostimulatory effect of *β*-glucans [12]. Ovarian cancer patients receiving AbM in addition to chemotherapy were found to have an improved quality of life and a higher NK-cell activity than patients receiving only chemotherapy [13]. Our group has previously performed a number of* in vitro*, preclinical, and clinical studies using the commercial Japanese product, AndoSan, which contains 82% of AbM together with two other mushrooms,* Grifola frondosa* and* Hericium erinaceus*. An increased production of cytokines and chemokines was demonstrated after incubation with AndoSan in cultures with human monocytes, umbilical vein endothelial cells, and monocyte derived dendritic cells (MDDC) [14, 15]. Incubation with AndoSan in MDDC cultures was shown to increase the expression of cell surface markers associated with activation and antigen presentation [16, 17]. Furthermore, in an mRNA assay on monocytic cells incubated with AndoSan, there was an upregulation of genes related to immune function, in particular in genes connected with proinflammatory cytokines [18]. In contrast, in patients with chronic hepatitis C receiving treatment with AndoSan, genes involved in apoptosis and cell proliferation were found to be upregulated but not genes related to immune function [19]. Oral intake of AndoSan was found to have an immunosuppressive effect both in healthy volunteers [20] and in patients with inflammatory bowel disease [21]. The reason for the difference between* in vitro* and* in vivo* effect on immune function may possibly be due to differential absorption from the gut of substances with immunomodulating effects [20]. An extended search in the Medline and PubMed databases failed to show any reports of toxic effects of AbM. Also, herb interaction studies with an* Agaricus* extract, later named AndoSan, demonstrated a very low inhibition of cytochrome P-450 metabolism (less than that for green tea), making clinically relevant adverse effects unlikely [22].

Based on the data cited above and* in vitro* experiments included in this report, showing an antiproliferative effect of AndoSan on mouse myeloma cells, we decided to investigate immunomodulating and clinical effects of AndoSan given as adjuvant therapy to patients with multiple myeloma scheduled to undergo high dose chemotherapy with autologous stem cell support.

## 2. Materials and Methods

### 2.1. Study Design

Patients with newly diagnosed multiple myeloma who had completed induction treatment and were scheduled to undergo stem cell mobilisation followed by high dose chemotherapy with autologous stem cell support at Oslo University Hospital, Norway, were eligible for the study. The patients received written and oral information about the study by the treating physician. Upon written consent, patients were randomized in a double blinded fashion to receive either AndoSan or placebo orally, 60 mL daily, starting from the day of stem cell mobilisation and continuing until the end of aplasia after high dose chemotherapy, a period of approximately seven weeks. Randomisation was performed by a study nurse drawing an envelope from a preprepared stack, containing a number from 1 to 50, in a random fashion. Each number corresponded to an allocated treatment (*Agaricus* or placebo), which was determined beforehand by a flip of a coin and known only to the study nurse. The study product (*Agaricus* or placebo) was prepared by the study nurse in identical dark glass bottles, identified only by the patient's study number. Thus, the content of the bottles was known to the study nurse but was blinded to the patients and the rest of the hospital staff.

The primary end points were (1) changes in serum levels of cytokines, chemokines, and growth factors in peripheral blood, (2) differences in expression levels of genes involved in immune activation by whole genome assay, both measured on the day of inclusion and at the end of intake of the study product, and (3) differences in the stem cell harvest product of a number of mononuclear cell subsets associated with the immune system. All biological samples were kept at −20°C and analyzed together at the end of the study. The blinding was unravelled after all laboratory tests had been performed. The secondary end points were (1) clinical response to treatment, (2) time in neutropenia, (3) days with body temperature above 38.0°C, (4) days with i.v. antibiotics after stem cell infusion, (5) time to new treatment, (6) overall survival, and (7) quality of life. The basis for the sample size (*n* = 40) was the results of two earlier studies showing significant changes in the levels of cytokines in healthy volunteers (*n* = 14) [20] and in patients with ulcerative colitis (*n* = 10) and Crohn's disease (*n* = 11) [21] after intake of AndoSan. The data were collected by the principal investigator and stored at a research file at the server of Oslo University Hospital. The study was approved by the Regional Committee for Medical and Health Research Ethics (REC South East). The Norwegian Medicines Agency was notified of the study according to national regulations.

### 2.2. Study Product

The commercial mushroom extract AndoSan, produced by the company ACE Ltd., Japan, and distributed by Immunopharma AS, Norway, was used as source of AbM. The extract contains the following Basidiomycetes mushrooms: 82.4% of AbM, 14.7% of* Hericium erinaceus, *and 2.9% of* Grifola frondosa*. AndoSan is registered as a food product in Japan, EU, and Norway.

Placebo was water with added color.

### 2.3. Chemotherapy

Stem cell mobilisation was induced by cyclophosphamide 2 g/m^2^ i.v and G-CSF. Stem cell harvesting by leukapheresis was started when the CD34+ cells in peripheral blood were >20 × 10^9^/L, that is, on days +10–+12 after stem cell mobilizing chemotherapy. Aliquots of the leukapheresis product were kept frozen and analysed by flow cytometry at the end of the study. High dose chemotherapy was melphalan 200 mg/m^2^ i.v. on day +2, followed by reinfusion of autologous stem cells on day 0 [23].

### 2.4. Proliferation Assay

A ^3^H-thymidine incorporation assay was performed on MOPC315.BM cells, that is, mouse myeloma cells [24]. The cells were suspended in RPMI 1640 (Lonza, Verviers, Belgium) supplemented with 10% fetal bovine serum (Sigma-Aldrich, Diegem, Belgium) and 1% Penicillin/Streptomycin (Sigma-Aldrich), plated in 96-well plates (5 × 10^4^/100 *μ*L/well) and cultured for 24 hours at 37°C and 5% CO_2_ in the presence of different AndoSan concentrations. For the last 16 hours of culture, 0.17 *μ*Ci of [Methyl-^3^H] thymidine (Perkin Elmer, Zaventem, Belgium) was added to each well. DNA was harvested on Multiscreen Harvest Plates (Millipore, Carrightwohill, Cork, Ireland) using Filter Mate Harvester (Perkin Elmer). Plates were dried for 3-4 hours before adding 25 *μ*L/well of Microscint O (Perkin Elmer) followed by radioactivity measurement (c.p.m.) with TopCount NXT Microplate Scintillation Counter (Perkin Elmer).

### 2.5. Quantitation of Cytokine Levels

The following cytokine, chemokine, and growth factor serum levels were measured at the day of inclusion and 1–3 days after the end of the intake of AndoSan, using multiplex bead-based sandwich immunoassay technology (Bio-Rad Laboratories AB, Sundbyberg, Sweden), strictly following the manufacturers instruction: IL-1*β*, IL-1ra, IL-4, IL-5, IL-6, IL-7, IL-8, IL-13, Eotaxin, G-CSF, IFN-*γ*, IP 10, MCAF, MP1-*α*, MP1-*β*, PDGF, RANTES, and TNF-*α*.

### 2.6. Identification of T-Cell Subsets, NK-Cells, and Dendritic Cell Subsets in the Leukapheresis Product

The leukapheresis products were suspended in RPMI 1640 medium (Life Technologies, Carlsbad, CA, USA) supplemented with 10% fetal calf serum and were analyzed using flow cytometry. Eight-colour analyses were performed for the identification of the lymphocyte subsets with the following monoclonal antibodies: CD2, CD3, CD4, CD5, CD7, CD16, CD25, CD56, CD45, CD45RA, CD45RO, CD9, CD127, CCR7, and HLA-DR antigens. All antibodies were purchased from BD Biosciences (San José, CA, USA) except for anti-CD8, anti-CD56, and anti-CD127 which were purchased from Beckman Coulter (Brea, CA, USA); anti-CCR7 was purchased from R&D systems (MN, USA) and anti HLA-DR from Biolegend (San Diego, CA, USA). The Blood Dendritic Cell Enumeration Kit (Miltenyi Biotech GmbH, Bergish Gladbach, Germany) was used according to the supplier's protocol to determine plasmacytoid dendritic cells, type 1 and type 2 myeloid dendritic cells. Flow cytometry analysis was performed on the LSR II instrument (BD Biosciences). Data analysis was performed using the Flow-Jo software (Tree Star, Ashland, OR, USA).

### 2.7. Gene Expression Studies

Bone marrow aspirate for gene expression studies was taken on the day of inclusion and 1–3 days after end of intake of the study product. Microarray analyses were performed using the Affymetrix GeneChip Human Gene 1.0 ST Arrays (Affymetrix, Santa Clara, CA, USA), which contains more than 28,000 gene transcripts. 150 ng of total RNA was subjected to Ambion WT Expression Kit (Ambion/Life Technologies, Carlsbad, CA, U.S.) and GeneChip WT Terminal Labeling Kit (Affymetrix, Santa Clara, CA, USA) following the manufacturers' protocols for whole genome gene expression analysis.

For comparison of gene expressions, a two-way ANOVA model was used. All raw intensities of microarray datasets were quantile normalized [25]. Genes with low expression variation, that is, maximum to minimum intensity less than twofold difference, were excluded leaving 7564 genes. Subsequently, a pair-wise Fisher's linear discriminant analyse [26] was used to select the top two percentages of the most differently expressed genes (i.e., 152 genes) between control and the* Agaricus* group. The selected genes were classified into four clusters by using* K*-means clustering algorithm [25]. In addition, microarray data were analysed through the use of Ingenuity Pathway Analysis (http://www.ingenuity.com/).

### 2.8. Clinical Data

#### 2.8.1. Classification

The patients were classified at inclusion according to the international staging system for multiple myeloma [27].

Treatment response was assessed by changes in the serum level of the M-component from start of induction until three months after high dose chemotherapy according to the international uniform response criteria [28].

*Time to regeneration of neutrophils* was the time between infusion of stem cells and the first day of a stable neutrophil count of 0.5 · 10^9^/L or above.

Time to new treatment was the time between inclusion and start of second line chemotherapy following progression or follow-up as of July 1, 2014.

Overall survival was the time between inclusion and follow-up as of July 1, 2014, or death.

#### 2.8.2. Quality of Life

Health related quality of life was measured at start of the study and three months after end of aplasia using the QLQ-C 30 questionnaire validated for multiple myeloma [29].

### 2.9. Statistics

Differences between changes in serum levels of cytokines, chemokines, and growth factor before and after treatment in the two treatment groups were calculated by the Independent Samples* t*-test on the IBM SPSS Statistics 21 program. The same program was used for calculation of differences in cell surface expression of leukocyte antigens in the leukapheresis product. The survival analyses were performed by the Kaplan-Meier test. Statistical methods used in gene expression analyses are reported under that section.

## 3. Results and Discussion

### 3.1. Myeloma Proliferation Assay

**Figure 1:**
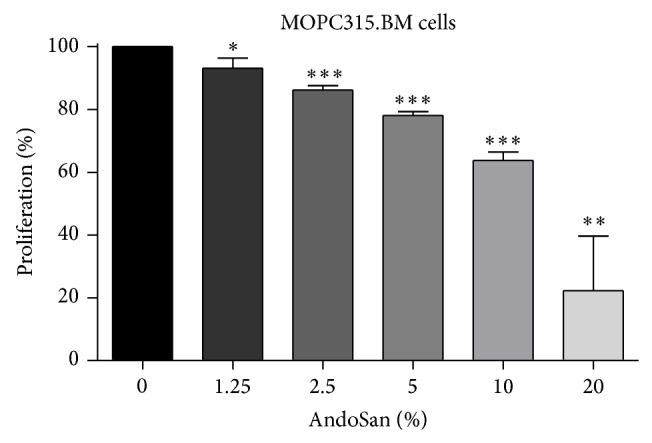
Effect of AndoSan on the proliferation of a murine multiple myeloma cell line* in vitro*. Proliferation of MOPC315.BM cells was assessed by ^3^H-labelled thymidine incorporation in the presence of different AndoSan concentrations (1.25%–20%). Results are expressed in percentage of proliferation (mean ± SD) relative to MOPC315.BM cells cultured without AndoSan (= 100%) and represent 3 independent experiments. Within each experiment, proliferation was assessed in triplicate. ^*^ *P* < 0.05, ^**^ *P* < 0.01, and ^***^ *P* < 0.001 (unpaired Student's* t*-test).

AndoSan significantly inhibited the proliferation of MOPC315.BM murine myeloma cells* in vitro* at a concentration of 1%. The observed inhibition was dose dependent (Figure 1).

### 3.2. Patient Number

From beginning of August 2009 until end of November 2010, 44 consecutive patients were invited to participate in the study, and 40 patients accepted. Nineteen were randomized to the* Agaricus* group and 21 to the placebo group. Three patients in the* Agaricus* group and four patients in the placebo group later withdrew from the study. Consequently, the total number of patients completing the study was 33, 16 in the* Agaricus* group and 17 in the placebo group. All included patients were evaluated for treatment response and survival, except for two patients with nonsecretory disease in each group who could not be evaluated for treatment response. Patients' characteristics are shown in Table 1. The inclusion was stopped according to schedule as the stipulated sample size had been reached.

### 3.3. Cytokines, Chemokines, and Leucocyte Growth Factors

A significant increase in serum levels of IL-1ra, IL-5, and IL-7 from inclusion until the end of intake of the study product was observed in the* Agaricus* group. No significant differences were seen in any of the other cytokines, chemokines, and growth factors (Table 2).

### 3.4. Cell Surface Markers in the Leukapheresis Product

Significantly higher percentages of Treg cells (CD4+, CD127d+, and CD25+) and plasmacytoid dendritic cells (CD303+) were noted in the* Agaricus* group compared to the placebo group (Table 3).

### 3.5. Gene Expression Studies

Gene expression studies were performed for eight patients in the* Agaricus* group and six patients in the placebo group at the time of inclusion and at the end of study. The selected differentially expressed genes were grouped in four clusters using* K*-means clustering algorithm. In cluster three are located several immunoglobulin related genes (IGKC, IgHV4-31, and IGKC) and genes related to Natural Killer cells, that is, Killer Immunoglobulin Receptors (KIR2DL3 and KIR2DL4). A low level of expression was noted for these genes in the control group and a high level of expression was shown in the* Agaricus* group (Figure 3). The Ingenuity system for phenotype-specific clustering of genes demonstrated upregulation of endosomatic HLA genes (Figure 4) and the plasma membrane CD86 gene (not shown) in the* Agaricus* group relative to the placebo group. Furthermore, this analysis showed a downregulation of IL-7 and CCL2 (MCP-1) genes in the* Agaricus* group relative to placebo group, whereas expression of IL-5 gene was unaltered (data not shown).

The records from the gene expression studies are registered in GEO (record number GSE 60869).

### 3.6. Treatment Responses

Treatment response could not be formally evaluated in two patients in each group because of nonsecretory disease. At inclusion, in the* Agaricus* group, 8/17 patients had reached at least partial remission after induction treatment, while the corresponding figure in the placebo group was 10/19. At the end of study, 16/17 patients in the Agaricus group and 18/19 patients in the placebo group had reached at least partial response.

*Median time to regeneration of neutrophils* was 14.2 days in the* Agaricus* group and 13.9 in the placebo group (n.s.).

*Days with temperature above 38.0°C* were 3.5 in the* Agaricus* group and 3.6 in the placebo group (n.s).

Days with i.v. antibiotics were 8.6 in the Agaricus group and 10.0 in the placebo group (n.s).

*Health related quality of life assessment* revealed no differences between the study groups (data not shown).

### 3.7. Survival

**Figure 2:**
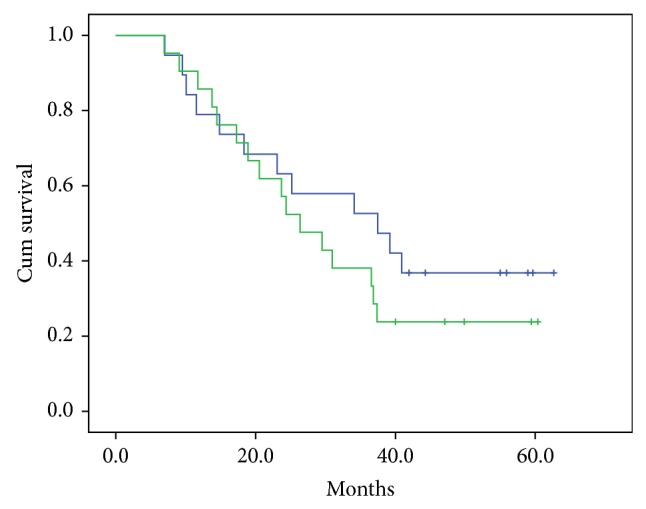
Time to new treatment. Mean time to new treatment in the* Agaricus* group (*n* = 19) was 37.3 months (upper (blue) curve) and in the placebo group (*n* = 21) 31.4 months (lower (green) curve) (*P* = 0.47 (n.s)).

At follow-up, 11/19 patients in the* Agaricus* group and 16/21 patients in the placebo group had started new treatment. In the placebo group, both patients with nonsecretory disease had started new treatment, whereas one of the two patients with nonsecretory disease in the* Agaricus* group had not. Mean time to new treatment was 37.3 months in the* Agaricus* group and 31.4 months in the placebo group (*P* = 0.49). Median observation time was 29.5 months (Figure 2) (*n* = 40).

At the same time point, 13/19 patients in the* Agaricus* group and 11/21 patients in the placebo group were alive. Mean overall survival was 50.7 months in the* Agaricus* group and 47.4 months in the placebo group (*P* = 0.93). Median observation time was 48.0 months (*n* = 40).

### 3.8. Discussion

A significant increase of IL-1ra, IL-5, and IL-7 serum levels was found in the* Agaricus* group compared to placebo, albeit the genes for IL-5 and IL-7 were found to be downregulated or unaltered, respectively, in the gene expression analysis. Also, the gene for the proinflammatory chemokine CCL2 (MCP-1) was found to be downregulated in the* Agaricus* relative to the placebo group.

*IL-1ra* is a natural inhibitor of the proinflammatory cytokine Il-1*β*, which serves a modulator for a variety of immune responses [30]. In particular, recombinant IL-1ra (Anakinra) is used in the treatment of rheumatoid arthritis and a variety of other autoimmune diseases [31, 32]. Elevated levels of IL-1ra have been documented in several types of cancer [33] including multiple myeloma, where it is associated with an improved prognosis [34]. IL-1ra counteracts IL-1*β*, which stimulates the production of IL-6 by bone marrow stroma cells. IL-6 is an important growth factor in multiple myeloma [35]. In a clinical trial, treatment with recombinant IL-1ra in patients with smoldering or indolent multiple myeloma was associated with a decreased myeloma proliferative rate [36]. On this background, the elevated levels of IL-1ra found in patients treated with AndoSan in our study may indicate a positive treatment effect of this product. This is also in line with the finding of reduced expression of the gene for the proinflammatory chemokine CCL2 (MCP-1).

The main role of* IL-5* is to stimulate the production of eosinophils [37]. It has recently been shown that eosinophils can stimulate the growth of malignant plasma cells [38]. The elevated levels of IL-5 found in the AndoSan group may therefore be interpreted as a negative factor, although there was an unaltered level of expression of the IL-5 gene. No clinical study on the role of IL-5 in multiple myeloma has to our knowledge been published.

IL-7 is a strong stimulator of both B-lymphocytes and T-lymphocytes [39]. In patients with multiple myeloma treated with high dose of melphalan with stem cell support, a gradual rise in plasma levels of IL-6, IL-7, and IL-15 was noted in aplasia, peaking on day +10 after infusion of hematopoietic stem cells [40]). It has been suggested that these cytokines may stimulate the proliferation of T cells in the autograft, among them also specific antimyeloma T cells [40]. In this perspective, the elevated IL-7 levels found in the AndoSan group may be interpreted as a positive treatment effect.

In the harvested stem cell product, increased percentages of regulatory T cells and plasmacytoid dendritic cells were found.

Regulatory T cells (Tregs) constitute a subpopulation of T cells, which modulates the immune system, maintains tolerance to self-antigens, and counteracts autoimmune disease [41]. Elevated levels of Tregs in peripheral blood have been found in both solid tumors and hematological malignancies [42, 43], including multiple myeloma, in which Tregs play a role in reducing immunosurveillance [44]. Excess of Tregs might result from the influence of inflammatory cytokines produced by tumor cells and tumor infiltrating lymphocytes [44]. In a clinical study on multiple myeloma, levels of Tregs were found to increase with increasing disease activity. Furthermore, high levels of Tregs were found to reflect a lower progression-free survival and total survival in patients treated with conventional chemotherapy. However, a predictive value for the levels of Tregs was not found in patients treated with high dose chemotherapy and stem cell support in this study [45]. In another study [46], the balance between suppressive Tregs and proinflammatory Th-17 cells was found to show a prognostic value for survival. In the present study, an increased portion of Tregs in the leukapheresis product was found in the* Agaricus* group compared to placebo. This may be interpreted as an immunosuppressive factor with negative impact on prognosis.

Plasmacytoid dendritic cells* (pDC)* stimulate both the innate and the adaptive immune system and induce tolerance. pDC levels are lower in multiple myeloma compared to control, the lowest levels being found in the clinically most advanced cases [47]. In the present study, proportions of pDCs in the leukapheresis product were higher in the patients who received AndoSan compared to control, which may suggest that AndoSan has a stimulatory effect, that is, a positive treatment effect, in these patients.

**Figure 3:**
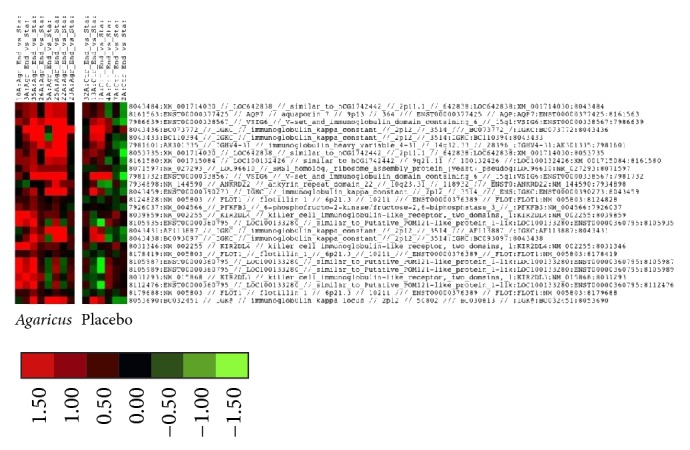
Gene expression analysis.* K*-means clustering algorithm. Cluster three. Several immunoglobulin related genes (IGKC, IgHV4-31, and IGKC) and genes related to Natural Killer cells, Killer Immunoglobulin Receptors (KIR2DL3 and KIR2DL4), are grouped together. These genes are more highly expressed in the* Agaricus* group (left column).

**Figure 4:**
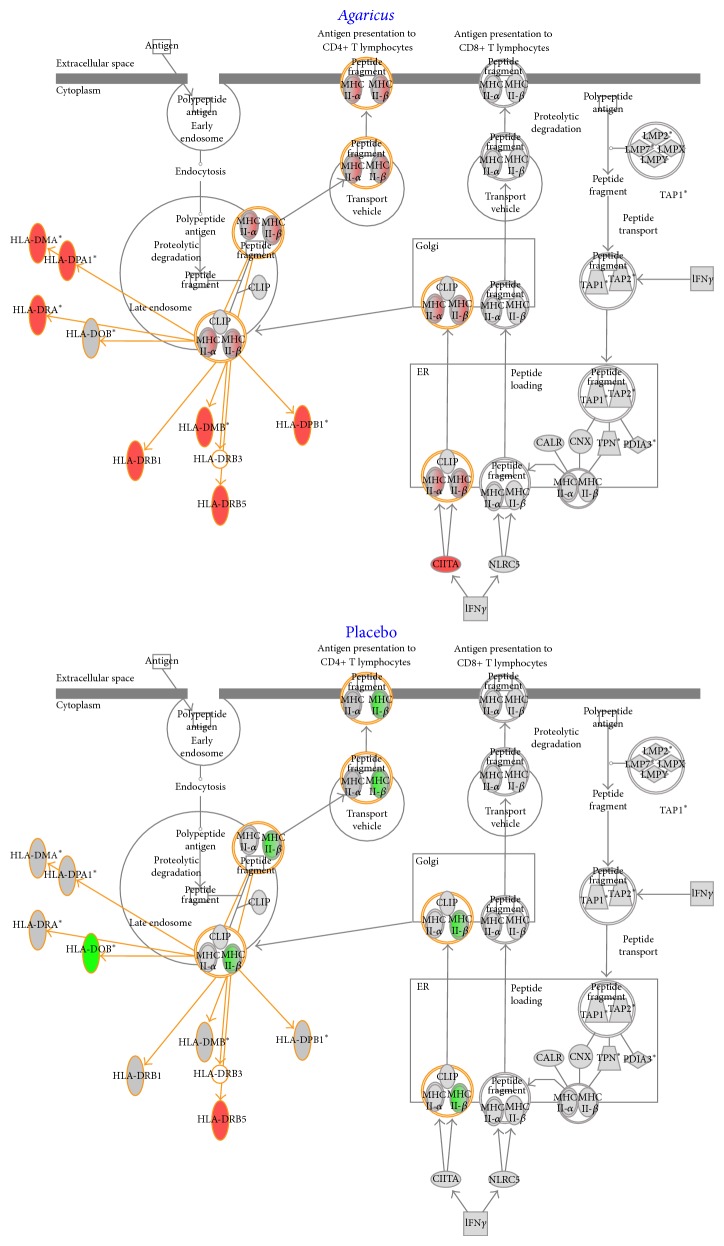
Ingenuity Pathway Analysis showing upregulation of genes in the HLA presentation pathway (symbols in red) in the* Agaricus* group and downregulation of HLA genes (symbols in green) in the placebo group.

Concerning the genetic analysis, an interesting pattern was revealed by* K*-means clustering algorithm. In cluster three, a number of immunoglobulin related genes (i.e., IGKC, IgHV4-31, and IGKC) and genes related to Natural Killer cells, Killer Immunoglobulin Receptors (i.e., KIR2DL3 and KIR2DL4), were grouped together. These genes had a low level of expression in the control group but were highly expressed in the* Agaricus* group suggesting an immunomodulatory effect of AndoSan (Figure 3). Furthermore, using the Ingenuity analysis system, an upregulation of HLA genes (Figure 4) and of the CD86 gene was found. In a previous study, we have found an upregulation of CD86, CD83, and CD80 on dendritic cells cultivated in the presence of AndoSan [15]. In the proliferation assay, a dose dependent inhibitory effect of AndoSan towards mouse myeloma cells was found, starting at 1%. This indicates that AndoSan may also have a direct antiproliferative effect on myeloma cells, which may be clinically significant. In earlier studies an inhibitory/tumoricidal effect of* Agaricus* has been reported in fibrosarcoma [5, 9] and ovarian cancer [6] and in human hepatocarcinoma [7] and leukemic cells [8].

A major difficulty in interpreting the results of this study is the fact that the composition of AbM and thus its presumed mechanism of action is at present unclear. Originally, it has been claimed from the producer that AndoSan contained 89% of carbohydrates, of which *β*-glucan constituted 28% [20]. However, a pharmacological analysis, which became publicly available after the completion of our study, showed that the net carbohydrate content in this product is considerably lower, that is, only 2%, corresponding to 0.09% of *β*-glucan. According to this analysis, the majority of the carbohydrates in this product consist of mono- and oligosaccharides [48]. The main mechanism of action of AndioSan may therefore be linked to other substances than *β*-glucans.

## 4. Conclusion

The study showed evidence of a number of immunomodulating effects of AndoSan, used as adjuvant therapy to high dose of melphalan with autologous stem cell support in patients with multiple myeloma, which possibly may have a clinical significance. However, the results must be interpreted with caution because of the restricted sample size of the study. No statistically significant clinical impact of AndoSan was detected, although trends for a longer median time to next treatment (37.5 months versus 31.2 months) and a shorter period of i.v. antibiotics (8.6 days versus 10.0 days) were noted in the* Agaricus* group. Further investigations of the effect of AndoSan in multiple myeloma in larger patient populations with a sample size large enough to detect clinical differences are needed in order to clarify whether AndoSan may have a role in the treatment of this disease.

## Figures and Tables

**(a):** 

Agaricus (*n* = 19)		
M/F	Age	Stage
M	65	II
M	56	I
M	36	I
F	61	I
M	63	I
F	65	II
M	59	I
F	64	II
M	56	III
M	46	III
M	62	II
M	66	I
M	65	I
F	48	II
F	49	I
M	64	I
M^*^	59	II
F^*^	59	II
F^*^	59	II

^*^Patients who withdrew from the study.

M/F = 12/7; mean age = 57,4; I = 9, II = 8, and III = 2.

**(b):** 

Placebo (*n* = 21)		
M/F	Age	Stage
M	56	II
M	42	II
M	55	III
M	61	III
M	61	II
M	62	II
F	44	III
M	60	II
M	56	II
F	62	I
M	58	II
F	56	II
M	52	II
M	61	I
M	55	I
M	52	II
M	51	I
M^*^	63	I
M^*^	63	II
F^*^	54	III
F^*^	52	III

^*^Patients who withdrew from the study.

M/F = 12/6; mean age = 56,6; I = 5, II = 11, and III = 5.

**Table 2:** Mean serum levels and range are shown for cytokines, chemokines, and growth factors (in pg/mL) at inclusion and at the end of intake of study product for the *Agaricus* (*n* = 16) and the placebo group (*n* = 17). The statistical relationship of the differences of the means of the two groups is shown in the column “A/P.”

	*Agaricus *			Placebo			A/P
	Start Mean	End Mean	Difference	Start Mean	End Mean	Difference	*P* value
IL-1ra	53.21	84.51	31.30	94.79	69.46	−25.33	*P* = 0.02
(Range)	(7.21–125.52)	(21.9–268.37)		(21.69–268.37)	(6.10–167.65)		

IL-4	1.57	2.01	0.44	1.82	1.84	0.02	n.s.
(Range)	(0.39–3.31)	(0.75–4.17)		(0.54–5.27)	(0.42–4.97)		

IL-5	1.66	2.89	1.23	2.75	2.21	−0.54	*P* = 0.05
(Range)	(0.02–4.89)	(0.19–9.37)		(0.36–8.29)	(0.43–5.60)		

IL-6	4.65	7.71	3.07	9.55	10.39	0.84	n.s.
(Range)	(0.03–11.32)	(0.88–18.40)		(2.29–28.67)	(2.60–32.82)		

IL-7	4.77	6.91	2.12	6.52	6.12	−0.40	*P* = 0.05
(Range)	(1.37–8.30)	(2.61–14.06)		(2.09–10.41)	(2.34–13.18)		

IL-8	12.59	15.14	2.54	13.49	15.41	1.91	n.s.
(Range)	(4.13–27.89)	(6.82–48.34)		(5.74–26.00)	(6.10–38.79)		

IL-13	5.58	5.98	0.40	6.78	9.27	2.49	n.s.
(Range)	(0.87–20.94)	(0.02–13.45)		(0.87–12.48)	(0.87–39.33)		

Eotaxine	93.11	105.31	12.20	162.33	112.39	−49.94	n.s.
(Range)	(0.11–247.02)	(16.59–229.25)		(23.02–600.07)	(11.00–368.73)		

G-CSF	19.06	21.94	2.89	22.34	19.54	−2.79	n.s.
(Range)	(5.60–41.66)	(9.63–38.64)		(9.99–34.41)	(7.82–33.92)		

gammaIFN	55.41	75.36	19.96	65.69	62.06	−3.62	n.s.
(Range)	(3.24–179.72)	(17.25–161.35)		(13.32–205.88)	(1.64–156.12)		

IP10	3198.89	3647.61	448.71	3230.03	3677.51	447.40	n.s.
(Range)	(1047.70–8564.00)	(391.37–12899.26)		(562.41–9314.66)	(277.79–13636.42)		

MCAF	63.73	43.46	−19.27	50.17	43.98	−6.20	n.s.
(Range)	(8.14–236.81)	(13.89–97.58)		(15.37–90.94)	(19.88–134.84)		

MIPa	50.46	42.48	−7.98	62.87	46.69	−16.16	n.s.
(Range)	(28.88–71.61)	(21.56–84.55)		(17.66–238.61)	(21.73–79.61)		

PDGF	426.31	450.75	24.44	454.23	*158.52 *	−295.71	n.s.
(Range)	(75.90–1327.12)	(19.06–3841.38)		(45.84–1632.87)	(20.99–309.16)		

RANTES	10217.89	7497.97	−2719.93	6496.38	4672.52	−1823.86	n.s.
	(1846.39–30848.81)	(1595.65–29043.80)		(644.32–23964.69)	(119.20–10435.40)		

TNF alpha	6.29	12.35	6.07	10.52	12.05	1.53	n.s.
	(0.97–21.66)	(0.22–42.11)		(0.97–41.87)	(0.22–50.85)		

**Table 3:** T-lymphocyte subsets and dendritic cell subsets.

	*Agaricus *	(*n* = 16)	Placebo	(=17)	*P* value
	Mean	Range	Mean	Range	
% of T-lymphocytes					
CD3+ T cells	86.2	(58.9–94.2)	86.7	(71.7–95.3)	n.s.
CD4+ T cells	50.8	(20.0–80.4)	51.8	(7.10–73.6)	n.s.
CD8+ T cells	40.4	(10.9–67.9)	39.6	(15.7–83.3)	n.s.
% of CD4+ T cells					
Naive (CD45RA+/CD27+)	24.7	(5.5–64.0)	29	(8.8–51.4)	n.s.
Central memory (CD45RA−/CD27+)	40.8	(20.3–72.3)	46.7	(30.0–73.5)	n.s.
Effector memory (CD45RA−/CD27−)	19.2	(3.1–37.2)	21.8	(5.5–38.8)	n.s
Terminally differentiated memory (CD45RA+/CD27−)	6	(0.3–38.5)	3.1	(0.2–9.4)	n.s
T reg = (CD4+/CD127d+/Cd25+)	11.8	(4.5–18.2)	9	(4.0–17.8)	*P* = 0.04
HLA-DR+ CD4+	29.6	(13.4–55.7)	26.6	(7.9–55.0)	n.s
% of CD8+ T cells					
Naive (CD45RA+/CD27+)	27.8	(2.4–69.9)	31.4	(5.9–62.5)	n.s
Central memory (CD45RA−/CD27+)	17.3	(1.5–32.8)	20.1	(3.2–43.5)	n.s.
Effector memory (CD45RA−/CD27−)	22.9	(4.4–51.7)	16.8	(4.6–49.3)	n.s.
Terminally differentiated memory (CD45RA+/CD27−)	32.8	(1.4–55.7)	31.7	(5.4–79.0)	n.s
HLA-DR+ CD8+	39.1	(7.5–71.6)	36.2	(5.9–69.3)	n.s
Others					
%NK cells (CD2 or CD7+ CD3−)	8.4	(0.9–35.8)	6.7	(1.7–26.7)	n.s.
% CD56b+	9.3	(0.7–35.5)	10.7	(0.1–32.7)	n.s.
% CD56b+ CD16+	70.6	(42.2–94.0)	69.4	(39.9–95.5)	n.s.
%CD56−CD16+	6.4	(0.1–34.1)	2.6	(0.1–15.3)	n.s.
%CD94+	65.5	(25.9–96.4)	60.8	(22.1–84.7)	n.s.
% of all cells except for CD14+ monocytes and CD19+ cells					
BDCA1 (CD1c+)	0.6	(1.0–1.3)	0.6	(0.1–2.4)	n.s.
BDCA2 (CD303)	1.1	(0.2–2.3)	0.7	(0.1–1.4)	*P* = 0.04
BDCA3 (CD141)	0.1	(0.04–0.4)	0.1	(0.03–0.3)	n.s.

The respective cell populations are given as frequencies of the cellpopulation to which it is a subset: T-cells and NK-cells as percentage of total lymphocytes; the major T-cell subsets (including CD4 positive, CD8 positive, and CD4/CD8 double negative or double positive); and NK cell subsets (including CD94 positive,  CD94 positive, CD56 bright positive, CD56 positive, Cd16 positive, and CD16 positive) as percentages of total T-cells and NK-cells, respectively: naive, central memory, effector memory, and terminally differentiated memory T-cells as well as CD4 positive T regulatory T-cells of CD4 and CD8 positive T-cells, respectively. The dendritic cell populations are determined within total cells excluding the CD14 positive and CD19 positive cells.
